# Enhancing adaptation and learning in educational environments (SENSE project): a case series study

**DOI:** 10.3389/fpsyt.2026.1810785

**Published:** 2026-05-08

**Authors:** R. Maggio, C. Pino, L. Catalfamo, M. A. Gregorio, A. Martina, G. Rao, M. Di Cara, G. L. J. E. Quattrocchi, F. Cucinotta

**Affiliations:** 1 IRCCS Centro Neurolesi “Bonino – Pulejo”, Messina, Italy; 2 Center for Autism “Dopo di noi” Barcellona Pozzo di Gotto, Messina, Italy; 3 “Capuana” public state Comprehensive School, Barcellona P.G., Messina, Italy; 4 UOC Rehabilitation for Adults and Minors, Barcellona P.G., Messina, Italy

**Keywords:** autism spectrum disorder, inclusive education, multisensory environment, school-based intervention, sensory regulation

## Abstract

**Background:**

Autistic students face increased risk of school exclusion, often linked to insufficient accommodation for sensory and social challenges. The absence of structured sensory breaks contributes to stress, behavioral escalation, and removal from the classroom. Recent studies recommend dedicated spaces to support sensory regulation.

**Aims:**

This community-based case series examines the use of a multisensory room (MSE) in a public school as a structured mid-morning break to reduce sensory overload and improve engagement.

**Methods:**

Five autistic students attended regular MSE sessions during school hours. Teachers received brief training from an ADC-P. Sessions provided individualized sensory support and were conducted with a neuropsychomotor therapist. Feasibility, safety, usability, and behavioral changes were assessed through service metrics, systematic observation, and teacher questionnaires.

**Results:**

The intervention was feasible, safe, and easily integrated into school routines. Organizational aspects and teacher training emerged as primary challenges. All students showed a reduction in challenging behaviors and improved sensory regulation, along with increased requests for preferred MSE activities.

**Discussion:**

Findings suggest that integrating MSE use into daily school practice may enhance sensory tolerance and reduce challenging behaviors in autistic students. As the first report of MSE use for sensory discharge in a public school during school hours, these results, despite the small sample, support further research within inclusive education models.

## 1 Introduction

Access to education is a fundamental human right (1). Despite legal measures and political and social attention to these issues (2), many students with disabilities still experience exclusion from the educational setting (3)with well-documented negative consequences for educational trajectories and socio-emotional well-being (3–5). Specifically, autistic students remain at heightened risk of school exclusion due to a wide range of academic, social, and environmental challenges (6–9). Among the key risk factors identified, insufficient accommodation to address the sensory challenges experienced by autistic students plays a central role (10–12).

Sensory processing alterations are a core and pervasive characteristic of Autism Spectrum Disorder (ASD) (13, 14), impacting 69%–95% of autistic people (14–16), and substantially influence how autistic individuals perceive, interpret, and interact with their environment (17).

Sensory differences in autism may involve all sensory modalities, including vision, audition, touch, smell, taste, proprioception, and vestibular processing (18). While sensory processing difficulties are common in autism, behavioral sensory profiles can vary widely across individuals (15, 19–21), as well as difficulties in integrating multimodal sensory information (15, 22). Furthermore, sensory atypicalities often involve multiple sensory domains simultaneously (23) and frequently co-occur and cluster in mixed sensory profiles that require careful assessment and individualized management (24). Since there are many variations in sensory responsiveness, educators need to be aware of their students’ specific sensory needs before the school year begins so that they can adapt the school environment to meet those needs (25). Indeed, sensory processing differences appear to be pervasive in everyday life, influencing development and adaptive functioning in both autistic children and adults (23, 26).

According to Dunn’s sensory processing framework (27–29), individual sensory experiences are shaped by the interaction between neurological thresholds for sensory input (high vs. low) and behavioral self-regulation strategies (passive vs. active). This model accounts for the wide heterogeneity of sensory-related behaviors observed in autism, such as hyper- or hyporesponsivity to sensory stimuli and active modulation strategies (e.g., avoidance or seeking) as adaptive responses shaped by underlying thresholds and regulatory strategies. In particular, heightened sensory sensitivities may lead to marked distress, social withdrawal, and significant impairments in daily functioning and social participation, thereby constituting a substantial barrier to inclusion and full participation in the educational and social contexts (30).

In educational contexts, sensory challenges can substantially interfere with comfort, participation, and academic performance (31–33). These individual differences become especially relevant in school settings, where sensory demands are continuous and frequently unpredictable and lack adequate support in mainstream settings (34). Ashburner and colleagues (35) have verified how a discrepancy between sensory needs and the school environment can significantly affect academic progress and results in most autistic children. Difficulties in sensory integration can adversely affect adaptive behavior, joint attention (20), motor coordination (36), emotion dysregulation (37), learning processes, reading and mathematical abilities (38, 39). Moreover, empirical evidence further indicates that students who experience difficulties in modulating sensory information from the environment often struggle to attend to stimuli that are relevant for learning (40–42). Finally, difficulties due to sensory disturbance and interpersonal modulation can negatively affect academic performance and classroom engagement, underscoring the importance of environmental adaptations and educational accommodations to facilitate learning (43, 44). All together, these observations highlight the need for feasible and individualized sensory support. Consequently, teachers and school staff are increasingly called to implement inclusive practices and develop sensory-friendly environments that support both learning and social participation of autistic students (45, 46). Although classroom-based sensory strategies are widely used, the classroom environment remains inherently dynamic and difficult to control, with multiple and unpredictable sensory stimuli. This may limit their effectiveness for students with pronounced sensory sensitivities, who may benefit from more structured and controllable environments (47, 48).

Interventions aimed at regulating sensory stimulation in everyday environments have shown beneficial effects for both autistic children and adults, including improvements in self-regulation, engagement, and behavioral outcomes (49–51). Within the field of sensory interventions, a distinction is made between Ayres Sensory Integration^®^ (ASI), a manualized and evidence-based approach, and sensory-based interventions (SBIs), which aim to modulate arousal through sensory input but currently show emerging and not yet conclusive evidence (48) Building on this evidence, several approaches have been proposed to support autistic students within school settings. One of the most inclusive solutions may be the creation of sensory-adaptive environments (SAEs). This term referred to the opportunity of adapting shared settings to sensory needs through technological and architectural modifications to reduce sensory overload (52). Multisensory environments (MSEs) are generally conceptualized as SBIs, as they provide controlled sensory stimulation without adhering to ASI principles. However, their implementation is often constrained by high costs and limited opportunities for individualized tailoring to each student’s sensory profile. Recent reviews highlight an increasing body of evidence supporting the use of structured sensory breaks to reduce sensory overload and behavioral dysregulation in students, while also pointing to persistent challenges in their consistent implementation within school settings (53–55). These findings are consistent with both vigilance and cognitive load theory. Taking a complete break from task-related processing enables cognitive resources to recover, thereby maintaining vigilance (56, 57). According to cognitive load theory, when the demands of perception increase, more resources are allocated to achieving top-down goals. Consequently, fewer resources are available for processing task-related stimuli [Lavie, 1995; Lavie, Hirst, de Fockert, & Viding,2004]. Investigating how different types of break affect vigilance performance could clarify the nature of these resources, even when sensory processing is involved. Moreover, preference of autistic people for environmental predictability is often reported (35, 58),and lies at the core of many leading theories of sensory processing in autism, such as Bayesian and predictive coding theories (59).Using an MSE, through control of sensory equipment, might enable them to better predict their own sensory experiences, and reduce feelings of ‘sensory overload’ (59, 60). Notably, caregivers and students frequently report that, even when individualized education plans are in place, the absence of structured sensory breaks can lead to behavioral escalation and, ultimately, school exclusion (46).

A solution often used in various contexts, both clinical and educational, is the creation of a quiet room: a room available for a sensory break from group activities. This “escape” space is designed as a place of relief from excessive exposure to stimuli (61, 62). The quiet room has also been described as a space that provides a ‘calming effect’ when autistic children display disruptive behaviors due to fatigue, discomfort, or overstimulation (63) and may look like a mini Snoezelen Curriculum Room (62).

Multisensory environments (MSEs) represent a promising and increasingly adopted approach in this regard. MSEs are dedicated spaces designed to provide controlled visual, auditory, tactile, vestibular, and proprioceptive stimulation (64, 65) and are commonly implemented in special education settings. Beyond their therapeutic aims, sensory rooms may offer immediate benefits by promoting comfort, reducing anxiety, and supporting self-regulation through immersive sensory experiences (66, 67). Crucially, MSEs allow for a highly controlled environment in which sensory input can be adapted to the individual’s sensory profile, offering flexibility in addressing both hyper- and hypo-sensitivities across sensory domains (35).

Preliminary evidence suggests that MSEs may help regulate arousal, enhance engagement, and reduce aggressive or stereotyped behaviors in autistic individuals (68–70). However, since stereotyped behaviors may serve a self-regulatory function, the goal is not their indiscriminate elimination, but rather to evaluate their reduced frequency as a potential reduction in intensity and frequency as well as better support for context-specific regulation strategies context-appropriate regulation strategies. This intervention does not aim to reduce stimming behaviors; existing literature affirms that stereotypies can have a regulatory effect (71). According to self-reports from autistic people, stereotypies can alleviate feelings of anxiety or being overwhelmed (71, 72).This can be particularly helpful in environments where sensory overload is experienced: reportedly, engaging in stereotypies modulates overwhelming sensory stimuli (71, 72) In this sense, a reduction in the frequency of stereotyped behaviors is viewed as positive, although a direct correlation with sensory relief in MSE cannot be established. However, in line with this perspective, some studies have reported a reduction in the frequency of stereotyped behaviors following MSE intervention compared to control conditions, although the results remain preliminary (60).

Additional reported benefits include improvements in self-awareness, social interaction behaviors, communication and exploration (73), increased relaxation (74), greater modulation of sensory search behaviors (75) and a reduction in self-stimulatory stereotypies alongside increases in adaptive exploratory and social initiation behaviors (76). Moreover, Unwin and colleagues (2022) (60) demonstrated that granting autistic children greater control over sensory changes within MSEs might create more favorable conditions for learning. By reducing perceptual discomfort and enhancing predictability, increased control over the sensory environment may support behavioral regulation and optimize readiness for both educational and therapeutic activities (60). These effects are primarily achieved within the MSE, where sensory stimuli can be systematically structured, modulated, and individualized, providing a controlled and predictable context that differs from typical classroom settings.

Despite these promising findings, the current evidence base remains limited, particularly with regard to the systematic use of MSE in mainstream educational settings rather than clinical or specialized contexts. Further research is therefore needed to clarify their feasibility, effectiveness, and mechanisms of action within school environments, as well as their potential role in promoting inclusion and preventing school exclusion among autistic students.

The aim of the “Sensory Environments for Nurturing Student Engagement” (SENSE) community projects is to explore the feasibility, usability and usefulness of MSE as a structured sensory-based regulation tool for autistic students in a mainstream public school. Specifically, the study aimed to monitor the progression of mands by promoting and supporting the emergence of spontaneous communicative requests; to observe changes in the frequency and characteristics of problem behaviors, including aggression, stereotypies, and escape behaviors; and to examine the feasibility, safety, and usability of the intervention through a questionnaire administered to teachers. The timing and structure of the intervention were guided by practical considerations and existing literature on sensory regulation in school settings, which suggests that structured sensory input and scheduled sensory breaks can reduce stereotyped behaviors and support attention and participation in autistic students (70). The MSE was implemented during the mid-morning period, when cognitive and sensory demands typically increase, to prevent dysregulation and support classroom engagement. Sessions (30 minutes, three times per week) ensured regular access while minimizing disruption to instructional time. Access was flexible and did not replace inclusive activities, allowing students to temporarily withdraw when needed and return to shared participation.

## 2 Method

### 2.1 Study design

This exploratory study examined the feasibility and preliminary outcomes of implementing scheduled mid-morning multisensory breaks for five autistic students within a public school. The study was conducted in an Italian public school, characterized by a model in which autistic students attend regular classes with the support of special education teachers, without separate specialized classes. The school day takes place mainly in the morning and includes a recess for all students. In this setting, the participating students took part in regular activities and recess with their peers, but were able to take additional personalized breaks to access the multisensory room (MSE) as needed, and then return to class to resume academic activities. This study received approval from the Ethical Committee IRCCS Sicilia Centro Neurolesi “Bonino-Pulejo” (n. 07/25). This research project adopted a pre-experimental AB design (77, 78). The data were collected repeatedly: 1) nine observational sessions were conducted in the classroom (Baseline – Phase A); 2) five sessions were conducted in the MSE and in the classroom in the hours following MSE use (Phase B).

Data were collected on multiple occasions: 1) Phase A (baseline) consisted of nine classroom observation sessions without any exposure to the multisensory environment (MSE). 2) Phase B (Intervention) consisted of five sessions, all of which involved observations in the MSE and in the classroom. The timing of classroom observations corresponded to the timing of observations carried out in phase A. Classroom observations were conducted during regular school activities (e.g., lessons or group tasks) scheduled in the same time period across sessions. Given the natural variability of the school context, activities were not identical; however, they were broadly comparable in terms of structure and demands, allowing observation of behavioral changes across baseline and intervention phases.

### 2.2 Participants

Participants were identified through referrals from clinicians of the neuropsychiatry service of IRCCS Neurolesi “Bonino-Pulejo” (Messina) and UOC Rehabilitation for Adults and Minors, Cutroni Zodda, Barcellona P.G. (Me), Italy, among students in the “*Capuana*” public state comprehensive school in Barcellona P.G. (Me), Italy, from January 2025. The participants’ legal guardian provided written informed consent after an explicit explanation of the study’s objectives and procedures. Children between the ages of 6 and 11 were also provided with an age-appropriate assent form, in accordance with ethical standards and institutional policy, and their assent was obtained before participation.

Eligibility criteria included (1) diagnosis of ASD according to DSM-5 (2); age between 4 and 18 years (3); sensory modulation alterations and/or difficulties (4); absence of severe comorbidities, such as uncontrolled epilepsy or sensory disorders (5); consistent attendance at the school program for the total number of sessions planned; and (6) signed informed consent.

### 2.3 Setting

The intervention was carried out at “*Capuana*” public state comprehensive school in Barcellona P.G. (Me), Italy. The MSE used for this study consists of a single, open space with soft flooring and a wide central area, equipped with several sensory devices providing controlled and customizable sensory stimulation. The environment includes:

- Visual stimuli: image projectors, soft lighting, fiber optics, vibrating bubble tubes, interactive panels, multicolor LED lights, and mirrors that enhance visual effects.
- Auditory stimuli: interactive musical panels, relaxing music, and nature sounds to support auditory engagement.
- Tactile stimuli: vibrating waterbeds, soft cushions, a hugging chair, a ball pool, rubber flooring, beanbags, and various textured surfaces designed to provide rich tactile input.
- Olfactory stimuli: essential oil diffusers (e.g., lavender, chamomile, eucalyptus) to stimulate the sense of smell.

All devices are equipped with simplified controls that allow operators to manage the environment and adjust individual sensory stimuli. The system is designed to be user-friendly and flexible, enabling easy adaptation to different student needs and sensory profiles.

### 2.4 Intervention

A specific intervention plan was developed for each participant based on individual preferences, sensory profiles, and their respective Individualized Education Plans. The plan also considered each student’s communication skills and potential problem behaviors. Before the MSE sessions, all special needs teachers and neuropsychomotor therapists received training on the use of the MSE. The entire process was supervised by the behavioral analyst – psychotherapist (ADC-P), who provided teacher training and structured both data collection and intervention procedures. All sessions conducted in the school’s MSE were led by trained special needs teachers, supported by neuropsychomotor therapists who facilitated interaction and the implementation of specific procedures, particularly Mand Training.

Students accessed the MSE in the morning, with schedules defined according to individual needs and the school’s organizational structure. Five sessions lasting approximately 30 minutes each were scheduled over a period of three weeks. The primary aim of the intervention was to facilitate adequate sensory discharge for every student.

During the first two sessions, the initial 15 minutes were dedicated to pairing, also referred to as “pre-session pairing” (79), a widely recommended behavior-analytic practice aimed at building a constructive relationship and establishing a positive therapeutic climate when working with ASD students (79–81). The goal of pairing in applied settings is to help the student associate the therapist or teacher, teaching materials, and the overall therapeutic context with preferred items and activities (i.e., positive reinforcers). Through this process, the therapist/teacher —initially a neutral stimulus—becomes a conditioned reinforcer (82), and the teaching context begins to signal improvement rather than deterioration in conditions (83). During this phase, the child was allowed to explore the environment freely, becoming familiar with objects and activities available in the room (e.g., water mattress, ball pool, mirrors, lights, sounds, swing). When the student showed interest in specific activities or demonstrated the absence of problem behaviors, the last 15 minutes of the session were dedicated to familiarizing them with additional objects and ensuring full exploration of the MSE. This also allowed the child to understand which items or activities were relaxing and could support sensory modulation.

The following three sessions began with a shorter pairing phase (10 minutes). When the student approached the teacher or therapist, consistently accepted adult-delivered stimuli, and the adult was able to maintain motivation (e.g., through play on the swing or in the ball pool), the teacher gradually increased opportunities for spontaneous requests through the Mand Training procedure, thereby promoting spontaneous and functional communication.

Mand Training was implemented through naturalistic teaching strategies based on Natural Environmental Teaching (NET), which allows learning to occur while the student is engaged in enjoyable, self-selected activities. The goal of using Mand training is to teach the student to express their needs in a functional and intentional manner, promoting autonomy, functional communication, and improved behavior regulation. NET is characterized by the use of natural contexts to facilitate language development, leveraging the student’s interests to guide instruction (84). Sessions also incorporated Incidental Teaching procedures (85). Incidental Teaching is a naturalistic teaching approach, typical of Natural Environment Teaching (NET), which involves teaching through playful activities and non-formal contexts. During treatment, the adult monitors and supports the child, creating a learning opportunity, providing prompts if necessary, and reinforcing behavior with access to the desired activity or object. Within MSE, this fosters the development of communication and interaction in a natural and highly motivating context, based on the child’s interests.

The 20 final minutes of the last four sessions were personalized to each student’s sensory preferences and communication abilities. At the same time, activities respected and supported each child’s sensory modulation and self-regulation processes.

At the end of each session, students were verbally prepared for the transition back to class. This preparation was tailored to individual needs. This procedure was implemented to minimize the occurrence of problem behaviors during transitions.

### 2.5 Measures

#### 2.5.1 Descriptive assessment

Each child has undergone an initial assessment to verify sensory preference and profile, Intellectual Quotients (IQ), and autism severity levels. Standardized tests were used for each area; IQ was evaluated using the Leiter International Performance Scale—third edition (86); autistic behaviors were assessed using the Autism Diagnostic Observation Scales—second edition (ADOS-2) (87); Adaptive functioning was determined using the Vineland Adaptive Behavior Scales—second edition (VABS II) (88). Moreover, sensory difficulties were assessed with The Short Sensory Profile (SSP) (28), a 38-item questionnaire, completed by the caregiver. It serves as a screening tool for children to identify potential sensory sensitivities or differences across various sensory systems.

#### 2.5.2 Feasibility, safety, and usability measures

**Figure 1:**
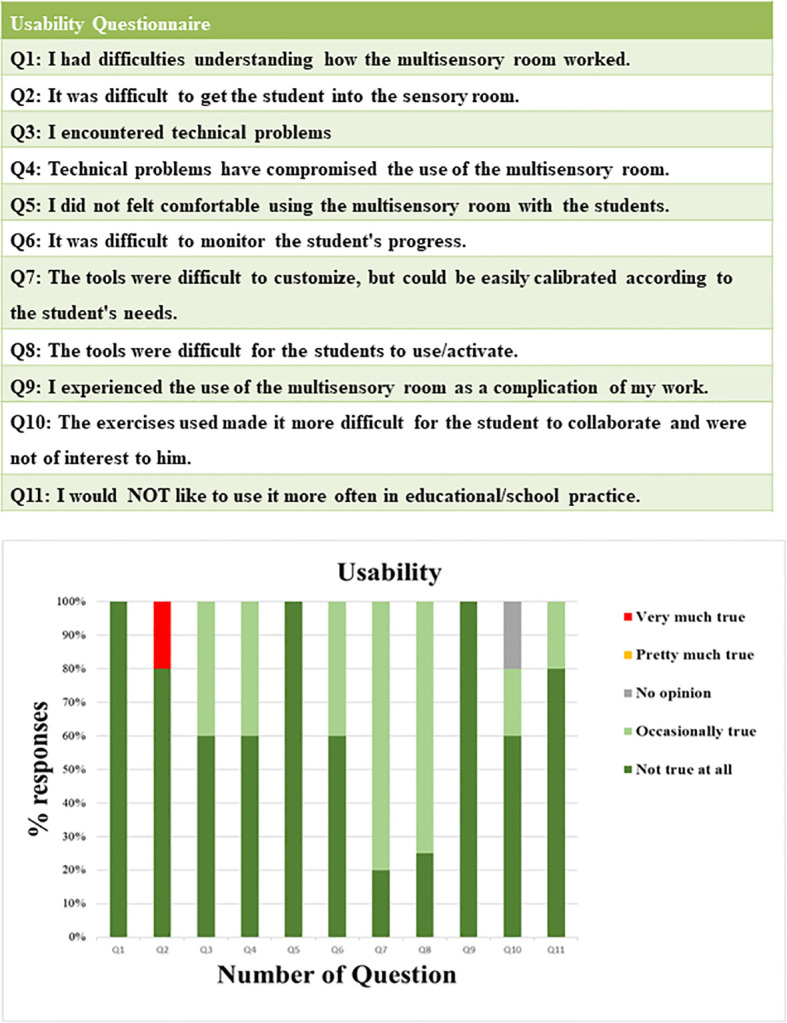
Usability questionnaire for teachers involved and results of usability questionnaires completed by each special needs teachers.

Feasibility was analyzed based on service utilization metrics, including participant adherence to MSE frequency and overall retention rate. Safety was assessed through systematic observation of participants’ responses during their experience in the MSE, with particular attention to signs of discomfort, fear, or other adverse reactions. To evaluate usability within the school context, teachers completed a usability questionnaire at the end of the intervention (see **Figure 1**). The questionnaire consisted of 11 items and used a 5-point rating scale, where 5 indicated “very true,” 1 indicated “not true at all,” and “no opinion” responses were scored as 3.

#### 2.5.3 Behavior coding

Data were collected on challenging or disruptive behaviors occurring daily in the classroom during school hours, as well as on positive behaviors exhibited during the students’ stay in the MSE. In all phases, observers recorded the duration of the sessions, the frequency of problematic behaviors, the communicative responses and the active collaboration of the participants.

The independent variable was access to MSE, including the frequency and duration of sessions, as well as the integration of mand training and incidental teaching procedures. Dependent variables included: challenging classroom behaviors (e.g., aggression, self-harm, highly intrusive stereotypies, and escape from task), spontaneous mands, eye contact, shared play, and sensory activity engagement, as well as measures of feasibility, usability, and safety (teacher questionnaire ratings).

Target behaviors were identified by a certified ADC-P, experienced in autism intervention, in collaboration with the special needs teachers assigned to each child. Daily data collection forms, completed by teachers and therapists, were used to record the frequency of problem behaviors, and/or motor/vocal stereotypies, and/or sensory-seeking behaviors. Data were collected both before the intervention (baseline), to inform planning based on observations in the natural context, and during the implementation of student-specific procedures (MSE period).

The ADC-P supervisor conducted two training meetings to instruct the special needs teachers and specialized educators on how to record data both in the classroom and during MSE activities. At the end of the training, ratter competency was verified through an inter-rater agreement test.

Multiple methods were used to ensure a comprehensive and detailed understanding of the context:

a) Qualitative tools, including regular interviews with teachers, to gather detailed information on student behavior and classroom dynamics, and to assess the sustainability of the intervention. These interviews were essential for continuous monitoring and included brief daily updates and more formal coordination meetings during crucial phases.
b) Quantitative tools, including systematic and structured direct observations, designed to measure and quantify specific student behaviors. These were implemented through predefined procedures and data collection instruments developed jointly by teachers and the ADC-P.

Across all phases, observers recorded session duration, frequency of problem behaviors, communicative responses, and participant cooperation. Specifically, problematic behaviors were recorded during class time, with observations conducted immediately before and after access to the MSE, in order to detect both the immediate effects of the sensory break and the return to normal teaching activities.

The ADC-P, together with each student’s educational team, selected the specific problem behaviors reported as interfering with learning to be monitored. Behaviors that could not be redirected or were not reported as interfering with learning were excluded. Additional challenging behaviors related to task avoidance (e.g., leaving the seat, self-distraction, throwing objects) and self- or other-directed aggression were also identified. In particular, problematic behaviors were recorded during class hours, with observations made immediately before and after accessing the MSE, in order to detect both the immediate effects of the sensory break and the return to ordinary teaching activities.

During the baseline period, each defined challenging behavior was coded for frequency during classroom hours by the trained special needs teacher, who also recorded the duration of each observation using a specific recording form (**Supplementary Figure 1**). The same procedures were used during the classroom hours of the MSE period, after the student began accessing the MSE.

During the student’s stay in the MSE, additional data were collected to identify the presence or absence of specific positive behaviors, including spontaneous or prompted requests (pure and impure mands), eye contact, shared play, reactions to aversive stimuli (unpleasant sensory activities), problem behaviors, and the types of activities engaged in within the MSE (e.g., water mattress, ball pool, swing, auditory or visual stimuli). These data were recorded by the neuropsychomotor therapist present in the room using a predefined form (**Supplementary Figure 2**).

Data analysis was performed primarily by visual inspection of the graphs, comparing the frequency and pattern of behaviors during the baseline period and the treatment phases. To assess procedural fidelity, interobserver agreement (IOA) was evaluated across all raters, yielding an average agreement of 93.6%. Dependent measures were assessed jointly by the supervisor and the specialized educator during observations conducted within the sensory room. For student behavior, IOA was calculated by dividing the number of agreements on checklist items (i.e., both observers marked “yes” for the same item) by the total number of agreements plus disagreements and converting the result to a percentage.

## 3 Results

### 3.1 Feasibility and safety

All children participated in every scheduled MSE session, resulting in a 100% retention rate. No adverse events, negative reactions, or signs of discomfort were observed at any point during the intervention. Safety was closely monitored throughout the study, with teachers and therapists systematically documenting participants’ behaviors and their responses to the activation of the various MSE devices. All participants well tolerated their time in the MSE, and no adverse events were recorded that raised safety concerns.

### 3.2 Sample characteristics and behavioral changes

The participants were five autistic children attending the “Capuana” public state comprehensive school in Barcellona P.G. (Me), of which four were male and one female. All participants had the support of a special needs teacher during school hours; in the Italian school system, these teachers assist the main teacher in the classroom by supporting children with special needs.

These students had similar levels of adaptive functioning skills, shared particular weaknesses in communication and socialization areas. The students’ difficulties in social interaction and communication were exacerbated by their inability to express their feelings and needs, leading to a lack of self-awareness and self-control. This led to frequent problem behaviors, some of which can be defined as severe due to their significant impact on the quality of learning of each student and overall on the quality of time spent within the classroom. In our description of patients, we will use fictitious names to protect their privacy better. Main sample characteristics were reported in **Table 1**.

**Table 1:** Sample characteristics.

Participants	Sex	Ethnicity	Age (yy)	Autism severity DSM-5	Intellectual quotient	VABS-II communication	VABS-II daily living skills	VABS-II socialization	VABS-II adaptive behavior	VABS-II composite score	ADOS 2 comparative Score	ADOS-2 classification
*Peter*	M	Caucasian	11	Lev.3	55	47	51	43	141	46	6	Autism
*William*	M	Caucasian	9	Lev.3	45	43	62	62	167	57	7	Autism
*George*	M	Caucasian	12	Lev.3	58	54	71	68	193	64	8	Autism
*Grace*	F	Caucasian	6	Lev.2	69	79	58	65	272	65	8	Autism
*Henry*	M	Caucasian	5	Lev.3	64	47	48	55	218	52	8	Autism

Student 1: Peter was an eleven-year-old autistic boy requiring Level 3 support, with a co-occurring mild intellectual disability (ID). He showed low adaptive functioning across all domains and limited use of verbal language, which he mainly employed to request basic needs or preferred activities. He exhibited significant distractibility, restricted and stereotyped interests (e.g., videos and moving objects), as well as immediate and delayed echolalia. No self-directed or other-directed aggression was reported. According to his family, he displayed heightened sensory impairments particularly in the gustatory/olfactory and visual/auditory domains. In the school context, Peter demonstrated marked difficulties with emotional and behavioral regulation, strongly influenced by his sensory profile. Monitoring data showed that, prior to exposure to structured sensory activities, Peter frequently engaged in escape behaviors and verbal protests. Transition periods or non-preferred demands often functioned as critical moments, during which motor agitation, kicking, sudden body dropping, and verbal refusals (e.g., “I don’t want it,” “I don’t like it”) were commonly observed. These episodes typically lasted between 5 to 20 minutes and occurred predominantly at the start of academic activities or in the presence of environmental stimuli perceived as intrusive.

The SSP indicated alterations in tactile, auditory, and gustatory/olfactory domains, suggesting difficulty in modulating internal sensations and external stimuli. Qualitative observations confirmed these findings: during daily activities, Peter seemed to struggle with unexpected noises, rapid environmental changes, or unintentional physical contact, which frequently preceded escape behaviors or vocalizations of protests.

**Figure 2:**
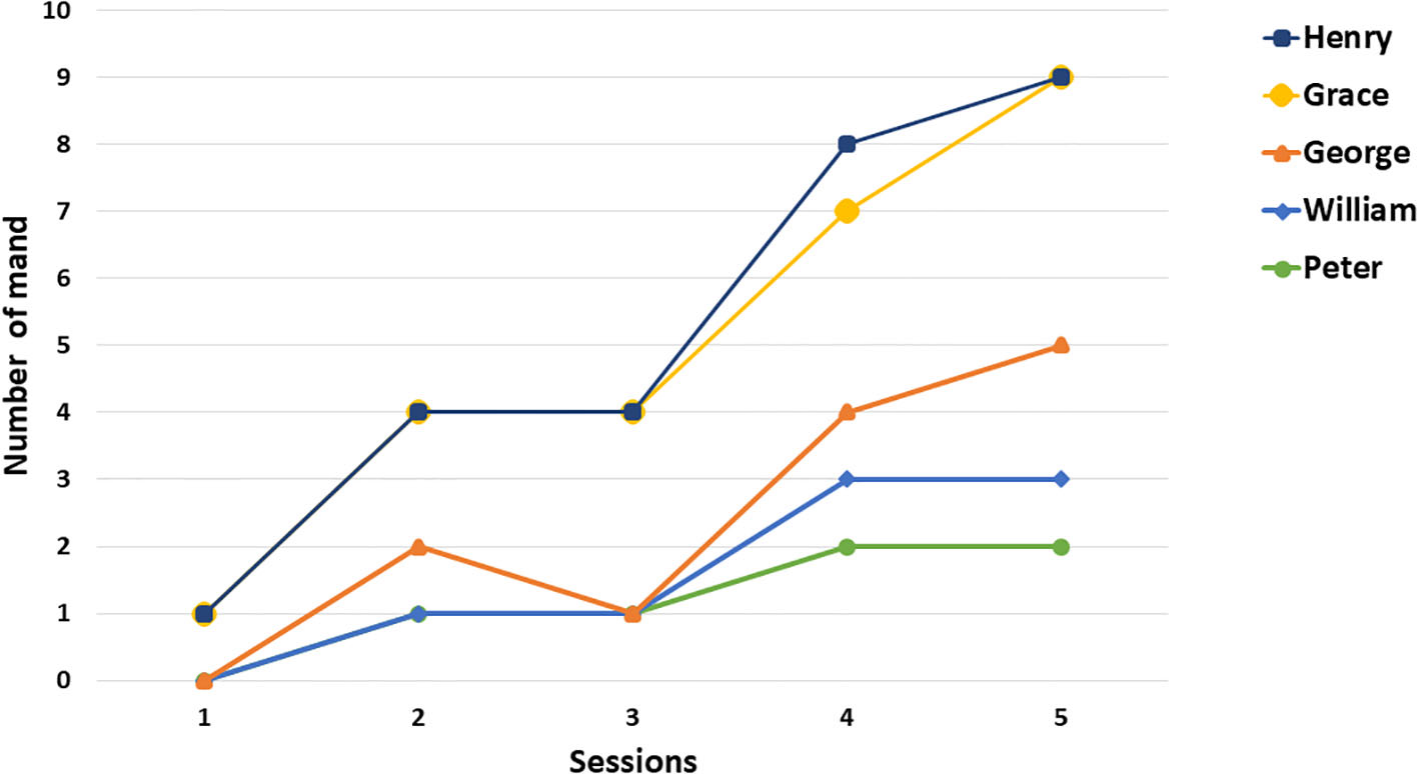
Spontaneous requests emitted by students in the MSE.

**Figure 3:**
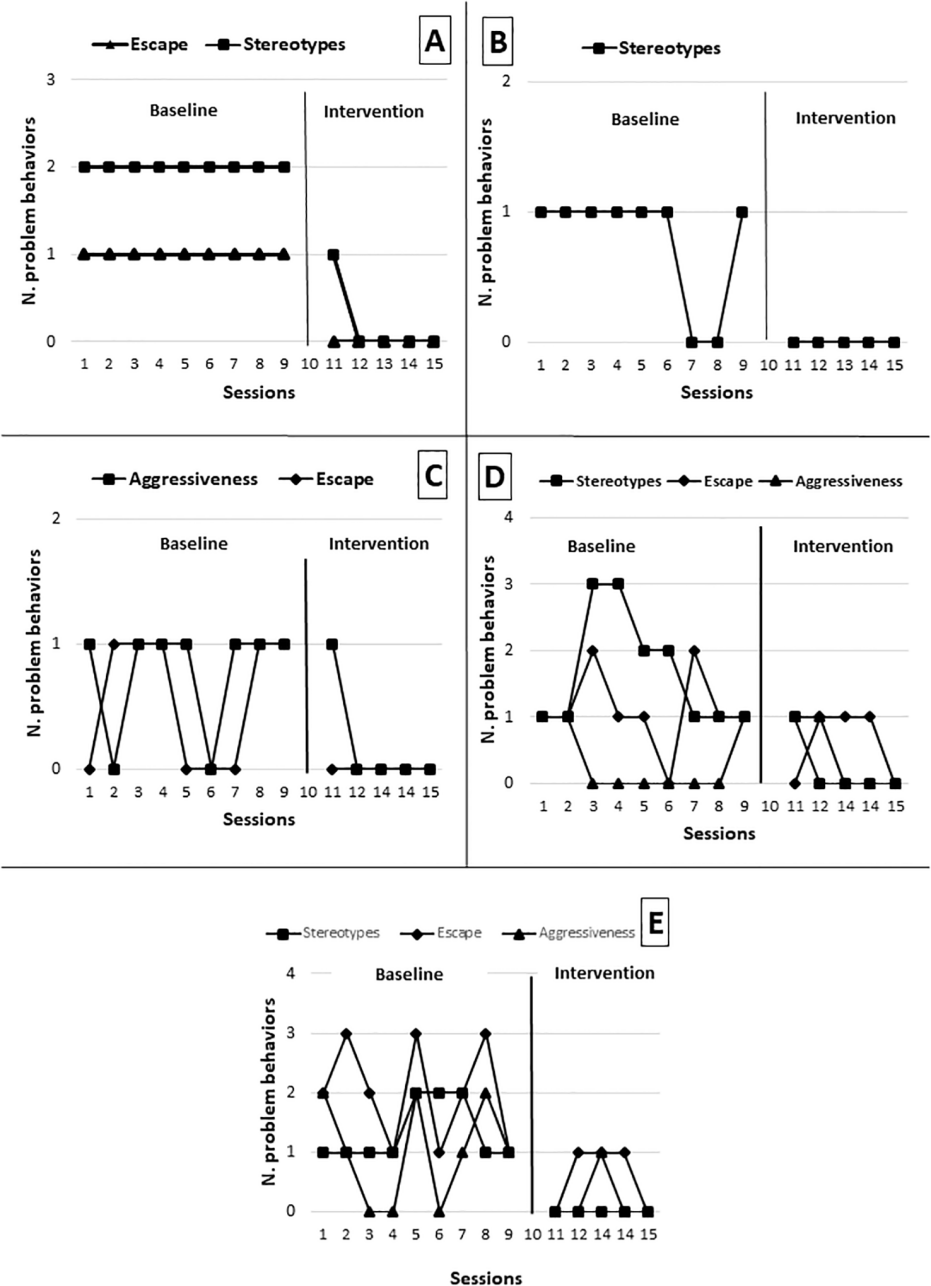
Frequencies of challenging behaviors in classroom; Panel **(A)** for student 1, Panel **(B)** for student 2, Panel **(C)** for student 3, Panel **(D)** for student 4, Panel **(E)** for student 5.

A significant improvement was observed each time Peter interacted with the MSE. In particular, the use of mirrors, illuminated surfaces, and rhythmic movement activities resulted as an enjoyable activity. The mirror was especially regulating: Peter would observe his face and movements, focus his attention, slow down his body rhythm, and appear more grounded and receptive. This type of predictable, controlled stimulation appeared to function as a genuine multisensory regulation experience, reducing arousal and restoring behavioral stability. Following exposure to controlled sensory stimuli in MSE, Peter generally appeared more oriented, more tolerant of adult guidance, and occasionally more willing to participate. The teacher reported that Peter, after engaging in highly motivating sensory activities (e.g., mirror interaction, manipulation of visual materials), he was able to remain on task for longer periods and showed fewer explosive reactions during transitions. Although there are difficulties in spontaneous communication, after sensory involvement in the MSE there appears to be a slight increase (See **Figure 2**). In **Figure 3**, panel A were reported frequencies of challenging behaviors in the classroom during Phase A, when the student exhibited two types of challenging behaviors in the classroom: motor stereotypies and task avoidance (placing his head on the desk). Following the introduction of the intervention, the frequency of these behaviors decreased after the participant received treatment in the multisensory room.

Functionally, Peter benefited substantially from brief sensory-based activities in MSE. These activities acted as a “bridge” between disorganized behavior and greater participation, enabling him to approach school routines in a more adaptive manner. The educational environment therefore benefited from the consistent integration of structured sensory stimuli, visual anticipations, and regulatory breaks to support his emotional balance and reduce dysfunctional behaviors.

Overall, Peter presents a complex profile, yet one with strong potential—particularly when interventions are implemented within a predictable, targeted sensory framework capable of positively modulating his emotional and behavioral responses.

Student 2: William was a nine-year-old autistic boy requiring Level 3 support, with a co-occurring moderate ID. He showed low adaptive functioning across all areas. As he did not use verbal language, he communicated his needs through the Picture Exchange Communication System (PECS). He generally presented as passive and engaged in frequent motor stereotypies such as rocking and repetitive hand movements. He appeared uncooperative during educational activities and expressed dissent by whining. No self-directed or other-directed aggression was reported. According to his family, he demonstrated more pronounced sensory impairments in the gustatory/olfactory and visual/auditory domains.

In the school context, William exhibited a pattern of functioning strongly influenced by his sensory profile and by difficulties managing intense stimuli, changes, and unexpected demands. Monitoring data indicated that, prior to accessing the MSE, William often showed signs of disorganized activation: he appeared passive, showed poor initiative, and used PECS exclusively for basic needs. His behavior alternated between periods of apparent calm and episodes of whining, rocking, and repetitive hand movements. Academic demands, especially early in the day, were difficult for him to accept; he often appeared withdrawn or minimally cooperative, with an emotional tone fluctuating between irritability and a tendency toward isolation.

The SSP revealed significant alterations in the gustatory/olfactory and visual/auditory domains, indicating probable difficulties in sensory modulation. These aspects were also evident during observations: William could overreact to certain stimuli or, conversely, become “absorbed” by specific sensory inputs, engaging in self-stimulatory behaviors such as body rocking or repetitive object manipulation.

Despite the passive attitude observed in the school environment, William displayed great curiosity toward the lights and sounds present in MSE. Moreover, he developed a strong preference for the swing. The combination of controlled, luminous, and rhythmic stimuli, together with the possibility of oscillatory movement, appeared to offer a framework in which he could better modulate his body and emotions.

Progress notes indicate that, over the sessions, William achieved increasing levels of engagement, relaxation, and relational availability. Within this environment, he not only tolerated interaction but actively sought it, expressing it through nonverbal means: he looked toward the adult, waited, and showed intentionality in his movements; increasing in requests to continue preferred activities through the use of PECS (**Figure 2**). A clear improvement in self-regulation emerged, with the MSE providing a predictable and interesting setting that facilitated his emotional stability.

The change became even more evident after leaving the MSE. The teacher documented a significant increase in attention span during school activities and a reduction in oppositional behaviors. William appeared more willing to follow instructions and could participate in proposed activities for longer periods. The quality of adult–child interaction also improved: William accepted adult presence more readily, showing a posture that was less defensive and more open. **Figure 3**, panel B shows that the student exhibited problematic behaviors in classroom, such as frequent motor stereotypies. The graph shows that the number of such behaviors decreased after the use of MSE.

Overall, William’s profile suggests that the MSE may represents not only a moment of well-being but a genuinely regulatory intervention capable of positively influencing his overall functioning. Prior to access, William tended to show fragmented behavior characterized by passivity alternating with whining and stereotypies; after the multisensory experience, he presented as more centered and more willing to communicate. His educational pathway therefore requires the regular inclusion of sensory-based spaces and activities that can act as a “bridge” to enhance educational participation and strengthen relational engagement with adults.

Student 3: The third student was a 10-year-old autistic boy with co-occurring mild ID and hyperkinesia, requiring Level 3 support. George also showed significantly lower adaptive functioning than expected for his age. Nonverbal, he communicated his needs using the PECS. He exhibited pronounced motor hyperactivity associated with motor stereotypies such as rocking and repetitive hand movements. He also showed heightened frustration in response to routine changes or loud noises, displaying both self-directed and other-directed aggression. In the SSP, his family reported greater sensory impairment in the hyporeactive/sensation-seeking and visual/auditory domains.

In the school context, George presented a complex profile characterized by major self-regulation difficulties, high levels of motor activation, and significant challenges in managing environmental stimuli. Documentation from daily monitoring highlighted a functioning pattern strongly influenced by routine changes, environmental unpredictability, and specific sensory triggers—such as the arrival of school buses or sudden loud noises—which frequently preceded episodes of severe dysregulation.

In the minutes preceding structured activities or during the early part of the school day, George often appeared hyperactive, agitated, and difficult to contain. Observations consistently described escape attempts, screaming, and disorganized motor behaviors, such as sudden running down hallways or repetitive hand movements. In situations perceived as stressful or aversive, he could exhibit more intense behaviors, including self- and other-directed aggression, inconsolable crying, and compulsive attempts to bite objects.

His sensory profile suggested—based on several documented episodes—a combination of hyporeactivity and sensory seeking, alongside heightened sensitivity to visual and auditory stimuli. George alternated between seeking intense sensory input and demonstrating extreme vulnerability to loud or overstimulating environments. Before accessing the MSE, therefore, his behavior was often dominated by agitation, chaotic sensory seeking, and difficulty orienting toward tasks.

Within the MSE or during structured sensory activities (e.g., use of soft mats, rhythmic movement, reduction of external stimuli), George showed reduced hyperactivity, improved regulation, and greater tolerance of adult presence. These moments, intentionally integrated into his routine as “sensory breaks,” aimed to help him regain a more functional level of activation and prepare him for subsequent activities.

During these sessions, although some motor stereotypies persisted (e.g., rocking, hand flapping), they appeared less intrusive to learning and more integrated into daily activity. His attention became more sustained, escape attempts decreased, and George seemed to use the controlled sensory input as a means to regain grounding and containment. Sometimes, George’s spontaneous communication seemed to increase slightly in the MSEs with more frequent requests (**Figure 2**). **Figure 3**, panel C shows that the student exhibited two types of problem behaviors in classroom: aggression toward teachers and avoidance of homework, such as getting up from his desk. After the start of treatment, a decrease in these behaviors was observed following his participation in the multisensory room intervention. Specifically, only one aggressive behavior occurred across the five sessions in which he accessed the MSE.

Post-intervention observations indicated that, after sensory sessions, George was generally more manageable. Returning to class was often less conflictual, and his motor activation appeared more modulated. He remained on task for longer periods, accepted adult support more readily, and exhibited fewer episodes of intense frustration.

A routine structured to alternate academic activities with sensory moments had a positive impact on his regulation. Following sensory breaks, George was less prone to sudden outbursts, escape behaviors, or aggression; moreover, he seemed better able to tolerate transitions and ambient noise, although these remained critical aspects of his profile.

Student 4: Grace was a six-year-old autistic student with mild ID, requiring Level 2 support. Her adaptive functioning was higher than that of the other participants, and she used short verbal sentences to express her needs. She exhibited high motor hyperactivity associated with stereotypic behaviors such as running. She also showed significant frustration when transitioning to non-preferred activities, and episodes of both self-directed and other-directed aggression were reported. According to her family, she presented greater sensory impairment in the gustatory/olfactory and visual/auditory domains. In the school environment, Grace displayed a complex profile characterized by emotional dysregulation, stereotyped behaviors, high motor activation, and a reduced ability to cope with non-preferred demands or transitions. Daily monitoring indicated that her behavior varied considerably depending on the predictability of the context, and especially before and after exposure to controlled sensory stimuli.

Before entering the multisensory room or beginning academic activities, Grace typically presented a clear state of hyperactivation. Observations frequently reported episodes of running back and forth across the classroom, self-stimulatory behaviors (e.g., hand movements in front of her eyes, bringing hands to her mouth), verbal protests, and escape behaviors in response to teacher demands. During transitions she often appeared disorganized, resisting directives through whining, crying, or abruptly leaving the workspace.

Her language was characterized by immediate echolalia and the use of simple, often repetitive sentences, frequently employed to protest against activities she perceived as non-preferred.

Before the frequency in MSE, Grace had difficulties in self-regulation: her behavior was fast-paced, impulsive, noisy, and poorly task-oriented. The greatest difficulties emerged during situations requiring waiting, turn-taking, or unexpected changes in activity sequence.

Documentation related to her participation in the MSE indicated a significant shift in behavior. The structured, predictable environment enriched with modulated sensory stimuli appeared to provide a highly regulating context for her. Soft lighting, illuminated surfaces, low-intensity sounds, and tactile materials helped slow her body rhythm and modulate her level of activation.

Particularly noteworthy was her strong interest in the swing—an activity she sought spontaneously and during which she appeared more centered and relaxed. Rhythmic oscillation seemed to offer her an effective form of self-regulation, reducing impulsivity and supporting a greater openness to adult interaction.

During multisensory sessions, Grace exhibited a reduction in more intrusive motor stereotypies and tended to show more organized behavior. Interaction became smoother: she accepted adult proposals more easily, maintained more stable eye contact compared with activities in the regular classroom and increased spontaneous requests (**Figure 2**). Sensory experiences thus appeared to provide a privileged channel for reducing internal stress and stabilizing her behavior.

Returning to the classroom after multisensory sessions was generally smoother. Subsequent monitoring indicated a reduction in escape behaviors, a more stable emotional tone, and greater tolerance for short, simplified requests. Although some stereotyped behaviors persisted, Grace appeared more oriented and able to remain engaged in tasks for slightly longer periods.

On certain days, documentation reported reduced opposition during transitions and improved motor coordination during group activities. **Figure 3**, panel D shows that the student exhibited three types of challenging behaviors in class: aggression directed toward teachers, avoidance of homework (running into her room), and motor stereotypies. During the use of MSE, the graph shows a decrease in all behaviors; both aggression and avoidance of homework occurred less frequently. Overall, multisensory support seemed to have a significant impact on the quality of her school participation: following sessions, Grace was typically calmer, more available, and less inclined to impulsive or disorganized responses.

Student 5: Henry was a five-year-old autistic boy requiring Level 3 support. Difficulties in adaptive functioning were present, together with a co-occurring mild ID. The child occasionally used single words to express his needs. He exhibited high motor hyperactivity associated with motor stereotypies. Significant frustration emerged in response to non-preferred activities or changes in routine, often resulting in reactive self-directed and other-directed aggression. According to his family, greater sensory impairment was reported in the gustatory/olfactory and visual/auditory domains. In the school setting, Henry showed a profile characterized by elevated motor activation, difficulty remaining calm during transitions, and marked fragility in emotional and behavioral regulation. Monitoring data collected before sensory activities indicated that he frequently appeared agitated, restless, and prone to escape behaviors, especially when confronted with non-preferred demands or sudden changes in routine. Several reports described him attempting to run away, rushing toward the door, or engaging in aggressive behaviors such as kicking, pinching, or pushing when he perceived excessive pressure or emotional overload.

The teacher also indicated that before multisensory sessions, Henry often appeared overwhelmed by noise, peers’ movements, and environmental demands. His typical behaviors included motor stereotypies such as repeated hand flapping, running in circles, or throwing himself onto the floor. He showed a constant search for intense sensory input, moving objects, vibrating surfaces, which he used spontaneously as attempts at self-regulation. Verbal communication was limited; he relied on gestures, isolated vocalizations, or occasional single words. Interactions with adults were mediated by unstable emotional states: he sought proximity when distressed but resisted direct guidance when overly activated.

Upon entering the MSE, Henry demonstrated an immediate and noticeable shift in behavior. The environment, enriched with controlled and adjustable sensory stimuli, appeared to precisely meet his sensory needs. He showed strong interest in the swing, the waterbed, and the light projector, elements that served as key channels for self-regulation.

As soon as he entered the MSE, Henry spontaneously moved toward oscillatory–rhythmic activities. The swing became a calming tool, with rhythmic motion helping him find balance and roundedness. When lying on the waterbed, he relaxed completely, showing a marked reduction in impulsive motor activity. Visual stimuli from the projector also contributed to his focus: Henry watched the lights with interest, slowed his movements, and appeared more connected with his surroundings.

During the first two multisensory sessions, Henry exhibited increased challenging behaviors. These difficulties were attributed to a significant disruption of routine, as he was accompanied by his mother during the first session and experienced an unexpected change in his usual school schedule. After this initial adjustment phase, his responses progressively stabilized, and he began to benefit consistently from the multisensory intervention. During subsequent sessions, aggressive behaviors decreased, as did escape attempts. His tolerance of adult presence improved, and at times he actively sought regulatory contact—for example, approaching the adult, maintaining visual attention, or accepting light guidance when transitioning from one stimulus to another. However, spontaneous communication has not increased (**Figure 2**). The multisensory context served as a decompressed space where stimuli were predictable, gentle, and calibrated to his sensory profile.

The return to class after multisensory sessions consistently showed meaningful improvements. Henry appeared calmer, less impulsive, and more able to orient toward proposed activities. Although difficulties persisted during highly structured demands, he tolerated adult support more readily and exhibited fewer problematic behaviors in the immediate post-session period. **Figure 3**, panel E shows that the student exhibited three types of challenging behaviors in class: aggression directed toward teachers, escape from work (leaving the room), and motor stereotypies. A decreasing trend in these behaviors was observed after the student participated in the MSE intervention. Specifically, only one aggressive behavior occurred overall the MSE sessions, along with two escape behaviors from the classroom to avoid schoolwork.

### 3.3 MSE usability in a school setting

All teachers strongly disagreed with the statements presented in the Usability Questionnaire, indicating no significant difficulties or challenges in using the MSE (**Figure 1**). Specifically, they reported no trouble understanding the functionality of the room (Q1), getting students into the room, except for Henry’s teacher (Q2), or encountering technical issues (Q3). Moreover, teachers felt comfortable using the room with students (Q5), although they occasionally encountered some difficulties in calibrating the instruments to individual needs (Q7) and in getting students to activate them (Q8). Monitoring student progress (Q6) was not viewed as problematic. Additionally, teachers did not perceive the use of the MSE as a complication (Q9), nor did they find the exercises disruptive to student collaboration or interest (Q10). Overall, there was a unanimous preference for continued use of the room in educational practice (Q11).

## 4 Discussion

The impact of sensory processing differences on autistic individuals’ mental health and quality of life it’s very significant (89, 90), the growing body of research on autistic sensory processing is a welcome development. However, existing literature has predominantly focused on individual sensory characteristics (91), with comparatively limited attention to the sensory demands imposed by everyday environments. This represents a notable gap, particularly in light of consistent evidence that autistic people experience many common settings as overwhelming and restrictive to participation (92–96). To address these difficulties, the United Nations (2022) have called for the provision of accessible public spaces for all by 2030, requiring the balancing of different access needs. Currently, some recommendations are being published to help services become more accessible to autistic people, such as the ASPECTSS Design Index (97), or for neurodiversity in a broad sense, including legislation, such as the Design Standard on Neurodiversity and the Built Environment (PAS 6463) published by the British Standards Institution (BSI, 2022).

In addition to standards design, several initiatives have been explored to modify how spaces are used with the intention of making places more sensory-inclusive for autistic people, for example large supermarket chains have introduced ‘quiet hours’, in which sensory input is reduced (98) or quiet spaces in places with high sensory stimuli (e.g. football stadiums in the FIFA World Cup (99).

However, empirical evidence supporting these guidelines and initiatives is scarce (100). Indeed, a small body of literature dealing with autism friendly design (101–103), some of which focuses specifically on school and classroom design (104). Recommendations often focus on creating a clearly structured environment, with clear navigation and well-ordered spaces (101), as well as lowered lighting and dampened sound (105). It is necessary to expand research data in order to develop more effective guidelines and promote policy changes that are beneficial to people with autism not only for new building and spaces, but also how to adapt and make existing or pre-constructed spaces and settings usable. In this regard, the SENSE project explore the feasibility, usability and usefulness of MSE as a structured sensory regulation tool for autistic students in a mainstream public school.

Based on previous literature, our study aims to offer compensation strategies for the sensory needs of autistic children in previously constructed facilities, environments where children spend a lot of time and often experience intense sensory pressure.

There is moderate evidence suggesting that SBIs, such as sensory stimuli through materials, tools, and activities (e.g., weighted items, passive swinging) accessed directly by the child, can impact functional behaviors (106–110). SBIs aim to temporarily modify the physiological arousal level with the goal to improve participation in tasks. SBIs are commonly used to support participation in daily activities for individuals with sensory challenges. MSEs can be considered a form of sensory-based intervention, for which the current evidence base remains emerging rather than conclusive. Specifically, few studies using tools or multiple sensory techniques were organized in school setting within the routine of the classroom. Based on this preliminary evidence, our program provides the use of the MSE during the mid-morning break on the school day to relieve the perceptual discomfort, help regulate arousal, promote engagement, and reduce aggressive or stereotyped behaviors, by offering sensory stimuli tailored to each user’s profile and controlled by the children themselves.

Although there are few studies in the literature on the use of MSEs with ASD (70), Fava (2010) (111) and Kaplan (2006) (112) have shown a reduction in aggressive behaviors, especially in the hour following MSE use, a reduction in problematic sensory behaviors (75)and an improved ability to focus on specific task (113).

To our knowledge, this is the first study that explores the use of MSE in a public-school setting used as relief from sensory stimuli and a structured sensory regulation tool.

A critical issue that has emerged from the Literature concerns the adaptation of existing public buildings. Although considerable attention must be paid to the construction of new spaces in line with the principles of sensory design, it is equally important to consider how existing environments, particularly those reserved by architectural, economic, or institutional limitations, can be modified to better accommodate people with sensory difficulties. This gap is particularly relevant in educational settings, where most school environments are already in place and cannot be drastically redesigned. Therefore, it is necessary to consider how these spaces can be optimized to meet the sensory needs of autistic people. Our findings support an adaptive approach, in which targeted interventions can mitigate sensory difficulties and facilitate student engagement, where solutions involving total architectural restructuring are not feasible and sensory elements that are not strictly related to the physical building appear difficult to control.

The use of MSE a school setting was explored by (114) for mixed groups of students with and without disability. In this study, the authors suggest that MSE is suitable for students in a in primary education contexts and participants appear to be engaged in all activities with pleasure. In term of feasibility, all children completed assessment and attended every MSE session, yielding a 100% retention rate, and no adverse events or negative reactions were observed. Furthermore, its ease usability was supported by teachers’ reports. All teachers did not perceive the use of MSE as a complication, although some reported difficulties in directing students in its use. Finally, few studies have been conducted that propose adapting SAEs based on the child’s sensory needs and preferences, after specific assessment (115). Our protocol demonstrates the feasibility of sensory assessment aimed at personalizing stimuli even in a public school setting. Considering that sensory needs vary from person to person among autistic individuals, and even within the same individual over time (90) research must consider how adaptations can be personalized, rather than being “one size fits all” (116) (reinforcing the need to implement them in all contexts.

In terms of safety, few studies have monitored this parameter within MSEs (70). The data collected confirm that, although only few patients were involved, the use of MSE by teachers and students in a chaotic environment such as mainstream schools appears to be safe and reliable.

Our outcomes support reports from other studies indicating that MSE can be well tolerated and integrated into daily routines when appropriately supervised and structured (60, 70) even with those who require a high level of support. In fact, 4/5 of the students required level 3 support and presented co-occurrence of ID. The features of the population involved appear to be important as they fall within the neglected end of the spectrum, i.e., a sample that is still underrepresented in research studies (117) (We believe that this target population makes the data obtained more important in terms of representativeness and generalizability in order to develop a fundamental evidence base to guide our clinical, social, and policy practices.

All participants exhibited reductions in challenging or disruptive behaviors (motor agitation, stereotypies, escape behaviors, aggression) following MSE sessions. This aligns with the findings of a recent systematic review that identified reductions in aggressive and stereotyped behaviors among autistic children and adults using MSEs (70). Moreover, a controlled observational study with 41 autistic children aged 4–12 demonstrated that when participants had control over the sensory environment, they exhibited increased attention and decreased repetitive motor behaviors, vocalizations, and overall activity levels (60). In our study, similar effects emerged, particularly when children could engage with preferred sensory stimuli (e.g., swing, waterbed, lights), suggesting that personal agency over sensory input may be a key mechanism for modulation (60, 118). This supports the hypothesis that MSEs serve not just as calming spaces, but as individualized regulation zones that accommodate each user’s unique sensory profile. Beyond reducing challenging behaviors, we observed improvements in engagement, task participation, and disposition to interact — outcomes also described in earlier research. For example, a 2024 study combining sensory-room therapy with conventional interventions reported improvements in sensory challenges, motor skills, and increased participation in extracurricular and daily activities (119). These enhancements may reflect reduced sensory distress, which frees cognitive and attentional resources for social and communicative engagement, a mechanism that may be interpreted in terms of sensory processing and regulation (29, 120).

The use of MSE as “quite room” seems to aligns with prior research describing MSEs as safe leisure or therapeutic settings when staff are trained and environmental stimuli are well controlled (121, 122) These results suggest that even in resource-limited public schools, MSE interventions may be realistically implemented — especially when targeted training is provided to educators and therapists (123). In routine school contexts, where multiple students may require sensory support, access to the MSE is necessarily organized on a scheduled basis, positioning it as a complementary resource alongside classroom-based strategies. In fact, although the usefulness of modifying school structures to reduce sensory overload remains to be confirmed, controlling all sensory stimuli present in schools appears to be very difficult, if not impossible. Implementing compensatory strategies, such as the use of MSE in the morning routine, appears to be easily achievable for all children and easy to implement in many schools. A recent review underlined that while SAEs are becoming more common in educational settings, evidence remains inconsistent and high-quality research is scarce (70, 115). Our study contributes to closing this gap by demonstrating that MSE integration in a mainstream public school is not only feasible and safe, but also potentially beneficial for behavioral regulation and school participation in autistic children. If replicated and expanded, this model could support a shift toward more sensory-attuned, inclusive schooling environments, potentially reducing school exclusion risks related to unaddressed sensory needs (108, 109).

This study provides encouraging preliminary evidence regarding the feasibility, safety, usability and potential benefits of using a MSE in a public-school setting for autistic children with sensory needs. Although evidence remains limited, our findings expand recent research by applying evidence-based principles to everyday environment. It proposes a practical strategy for adapting existing spaces to better support autistic children’s engagement and participation in mainstream school, emphasizing the importance of translating research into feasible, real-world interventions.

## 5 Limitations and future directions

The main limitation of this exploratory study is the sample size that limits the generalizability of the findings. Nevertheless, this approach is particularly useful in behavioral research, as it allows functional relationships to be established between environmental conditions and the behaviors under investigation (124). The baseline data collection phase allowed us to establish existing levels/patterns of the behaviors of interest, enabling us to appreciate changes during the intervention phase and, overall, provided preliminary objective data on the feasibility of the planned intervention, which was necessary to propose its application to a larger number of students in a public school.

The study has other methodological limitations that must be considered when interpreting the results. First, data collection was carried out mainly by teachers, with the potential risk of observational bias. A further limitation concerns the absence of direct feedback from children regarding their enjoyment of using the MSE: assessments were based exclusively on indirect behavioral indicators, such as the presence/absence of signs of frustration or avoidance behaviors during their stay in the room. Other important variables were not controlled for, but could impact student behavior: the type of classroom activities undertaken after using MSE, and general characteristics of the classroom environment (e.g., noise, visual stimuli, changes in routine). These factors could influence the observed effect of MSE and should be considered in future research. Finally, long-term data were not collected, so it is not possible to assess the persistence of the effects of MSE on self-regulation skills and classroom engagement.

To address these limitations, future research should certainly consider expanding the sample to include a larger number of participants and different age groups in order to increase the generalizability of the results. It will also be necessary to integrate video recording, which would allow for more objective behavioral coding and the possibility of blind analysis. Notably, direct feedback from children on their enjoyment of using the MSE should be included. This is a crucial step in further optimizing the adaptation of spaces to their specific sensory needs, but also in allowing them to express their own perceptions, preferences for greater active participation in the design process. Future studies should also explore the transferability of selected MSE elements into classroom settings, particularly in contexts where dedicated spaces are not available. While full MSE installations may require substantial resources, simplified or portable sensory adaptations could represent a more accessible alternative. Additionally, investigating the feasibility of small-group use may provide further insights into how such interventions can be realistically implemented in diverse school contexts.

## Data Availability

The raw data supporting the conclusions of this article will be made available by the authors, without undue reservation.
