# Supplementary material for: Enhancing adaptation and learning in educational environments (SENSE project): a case series study
Source: Front Psychiatry. 2026 May 8;17:1810785. doi: 10.3389/fpsyt.2026.1810785 (PMC13194407; doi:10.3389/fpsyt.2026.1810785)

**Enhancing adaptation and learning in educational environments (SENSE Project): a case series study**

**Maggio R.^1,2^, Pino C.^3^, Catalfamo L. ^3^, Gregorio M.A. ^3^ , Martina A. ^4^, Rao G. ^4^, Di Cara M.^1^, Quattrocchi G. L. J. E.^4^, Cucinotta F^1^***

Supplementary Material

# Supplementary Figures


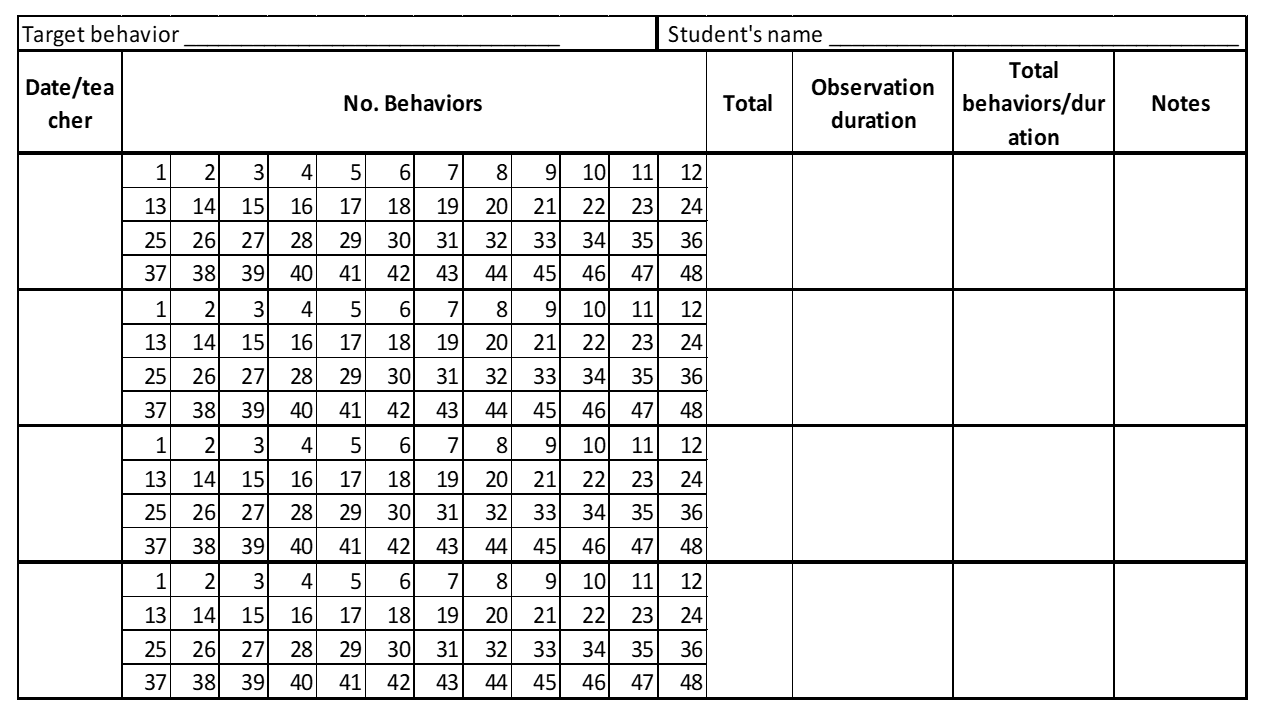


**Supplementary Figure 1.** Data collection form for in-class daily monitoring.

**Supplementary Figure 2.** Data collection form for in MSE monitoring


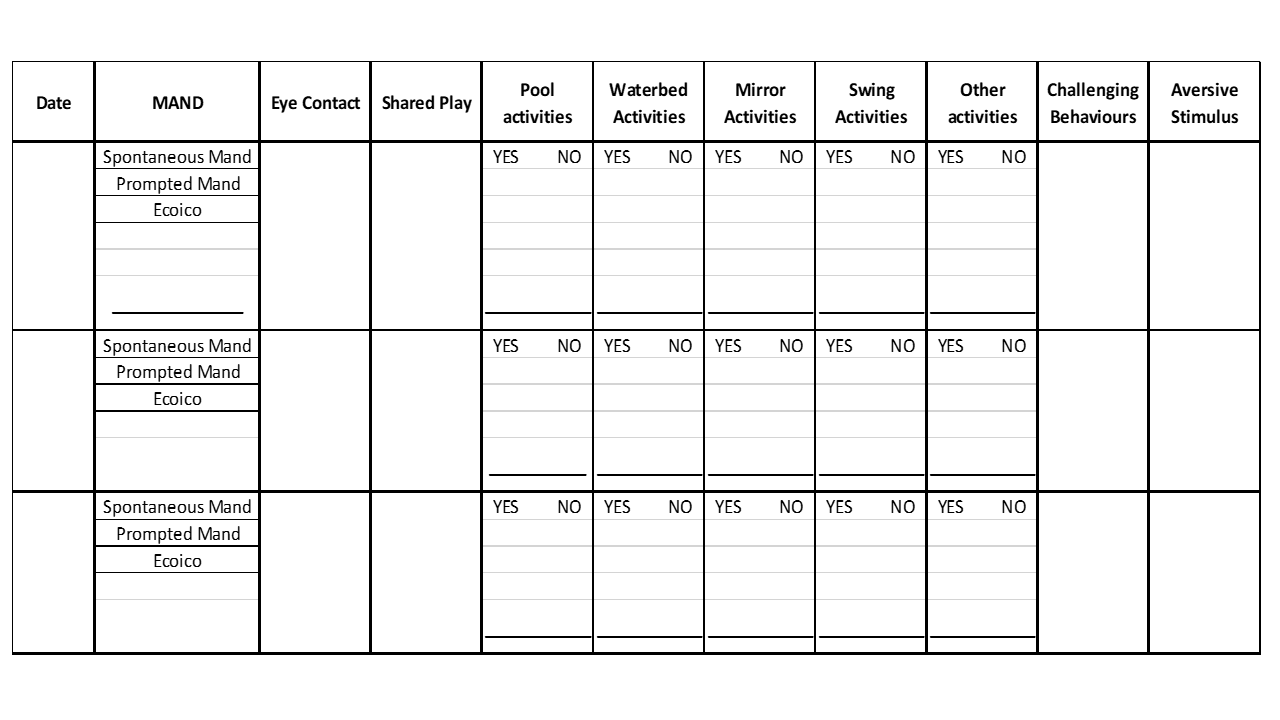

Supplement: Supplementary file 1 [file Table1.docx]
